# Guidelines for the functional annotation of microRNAs using the Gene Ontology

**DOI:** 10.1261/rna.055301.115

**Published:** 2016-05

**Authors:** Rachael P. Huntley, Dmitry Sitnikov, Marija Orlic-Milacic, Rama Balakrishnan, Peter D'Eustachio, Marc E. Gillespie, Doug Howe, Anastasia Z. Kalea, Lars Maegdefessel, David Osumi-Sutherland, Victoria Petri, Jennifer R. Smith, Kimberly Van Auken, Valerie Wood, Anna Zampetaki, Manuel Mayr, Ruth C. Lovering

**Affiliations:** 1 Centre for Cardiovascular Genetics, Institute of Cardiovascular Science, University College London, London WC1E 6JF, United Kingdom; 2 The Jackson Laboratory, Bar Harbor, Maine 04609, USA; 3 Ontario Institute for Cancer Research, Toronto, Ontario, M5G0A3, Canada; 4 Department of Genetics, Stanford University, MC-5477 Stanford, California 94305, USA; 5 Department of Biochemistry and Molecular Pharmacology, NYU School of Medicine, New York, New York 10016, USA; 6 College of Pharmacy and Health Sciences, St. John's University, Queens, New York 11439, USA; 7 Zebrafish Model Organism Database, 5291 University of Oregon Eugene, Oregon 97403-5291, USA; 8 Karolinska Institute, Department of Medicine, Center for Molecular Medicine (CMM) L8:03, Stockholm 17176, Sweden; 9 European Molecular Biology Laboratory, European Bioinformatics Institute (EMBL-EBI), Wellcome Trust Genome Campus, Hinxton CB10 1SD, Cambridge, UK; 10 Human and Molecular Genetics Center, Medical College of Wisconsin; 11 Department of Physiology, Medical College of Wisconsin; 12 Department of Surgery, Medical College of Wisconsin, Milwaukee, Wisconsin 53226, USA; 13 Division of Biology, California Institute of Technology, Pasadena, California 91125, USA; 14 Cambridge Systems Biology and Department of Biochemistry, University of Cambridge, Sanger Building, Cambridge CB2 1GA, United Kingdom; 15 King's British Heart Foundation Centre, King's College London, London SE5 9NU, United Kingdom

**Keywords:** annotation, biocuration, GO, microRNA, function, analysis

## Abstract

MicroRNA regulation of developmental and cellular processes is a relatively new field of study, and the available research data have not been organized to enable its inclusion in pathway and network analysis tools. The association of gene products with terms from the Gene Ontology is an effective method to analyze functional data, but until recently there has been no substantial effort dedicated to applying Gene Ontology terms to microRNAs. Consequently, when performing functional analysis of microRNA data sets, researchers have had to rely instead on the functional annotations associated with the genes encoding microRNA targets. In consultation with experts in the field of microRNA research, we have created comprehensive recommendations for the Gene Ontology curation of microRNAs. This curation manual will enable provision of a high-quality, reliable set of functional annotations for the advancement of microRNA research. Here we describe the key aspects of the work, including development of the Gene Ontology to represent this data, standards for describing the data, and guidelines to support curators making these annotations. The full microRNA curation guidelines are available on the GO Consortium wiki (http://wiki.geneontology.org/index.php/MicroRNA_GO_annotation_manual).

## INTRODUCTION

The past two decades of research have established that microRNAs (miRNAs) play a central role in regulating the stability and expression of messenger RNAs (mRNAs). These molecules are the focus not only of intensive basic research to better define their roles in regulating and integrating biological processes, but also of applied studies to exploit their potential value as biomarkers and therapeutic agents (Janssen et al. 2013; Caputo et al. 2015; Emanueli et al. 2015). MiRNAs are ∼22-nucleotide (nt) sequences that function by forming duplexes with their mRNA targets. The targeted mRNA can be regulated, or “silenced,” by a variety of mechanisms including reduction of mRNA translation, mRNA cleavage or promotion of mRNA degradation via deadenylation (Filipowicz et al. 2008).

MiRNAs have also been shown to activate a gene's expression (Zhang et al. 2014), although there is limited support for this and it remains to be seen whether this is a common event. More than 2500 mature human miRNAs have been identified so far according to miRBase assembly version GRCh38 (Kozomara and Griffiths-Jones 2014), each of which can target multiple mRNAs and can have different effects on different targets. MiRNA interactions can therefore potentially define networks of coordinately regulated genes and suggest novel molecular strategies for integrating and modulating cellular processes. Studies of miRNAs have been carried out by many independent groups using diverse experimental strategies, and integration of the resulting data is difficult. Even the identification of all mRNAs that can bind a given miRNA to form a stable duplex under physiological conditions is difficult given the range of approaches used and the variable data quality. This problem is reflected in miRNA databases where targets of miRNAs can frequently be reported based on weak or nonexistent evidence. For example, often the cited paper does not provide a rigorous experimental validation, or the miRNA:mRNA association is based on an unsound inference from a text-mining algorithm. These inaccuracies and weak inferences impede data mining and integration efforts (Kalea et al. 2015).

The Gene Ontology (GO) has been successfully applied to organize similarly complex catalogs of proteins and to impose quality, reliable standards on their annotation (Huang et al. 2009; Alam-Faruque et al. 2011; Mutowo-Meullenet et al. 2013). GO annotation condenses experimental data from peer-reviewed articles into a resource that is easily accessible by both scientists investigating small data sets and bioinformaticians performing complex computational analyses (Ashburner et al. 2000). An especially useful application of GO is functional analysis, or “GO term enrichment,” which can be used to identify pathways and processes that are significantly over- or underrepresented for a list of genes, e.g., differentially expressed genes from a microarray experiment (Huang et al. 2009). Currently, functional analyses of miRNAs or miRNA high-throughput data sets commonly use the GO annotations associated with the genes or gene products the miRNAs are predicted to regulate (e.g., see analyses in Liu et al. 2010; Soh et al. 2013). Since each miRNA may target up to several thousand mRNAs, each one potentially playing roles in multiple physiological processes, the interpretation of such analyses can be misleading. It was recently demonstrated by Bleazard and colleagues that terms enriched when using functional annotation of miRNA targets do not remain significant when target distribution bias is corrected (Bleazard et al. 2015). More informative results should be gained by using the experimentally validated functional annotations of the miRNAs in analyses. Here we describe the development of standards to ensure a consistent approach to the GO annotation of miRNAs and their targets. This includes the development of ontology terms to represent current knowledge, standards for describing the published data and guidelines to support curation of this data. The field of miRNA biology continues to develop very rapidly at both a technical and conceptual level; to accommodate this we have focused on a framework that will handle current data types and the development of open-ended and extensible standards appropriate for additional classes of molecules, molecular functions, and biological processes.

## RESULTS

### Ontology development

**FIGURE 1.**
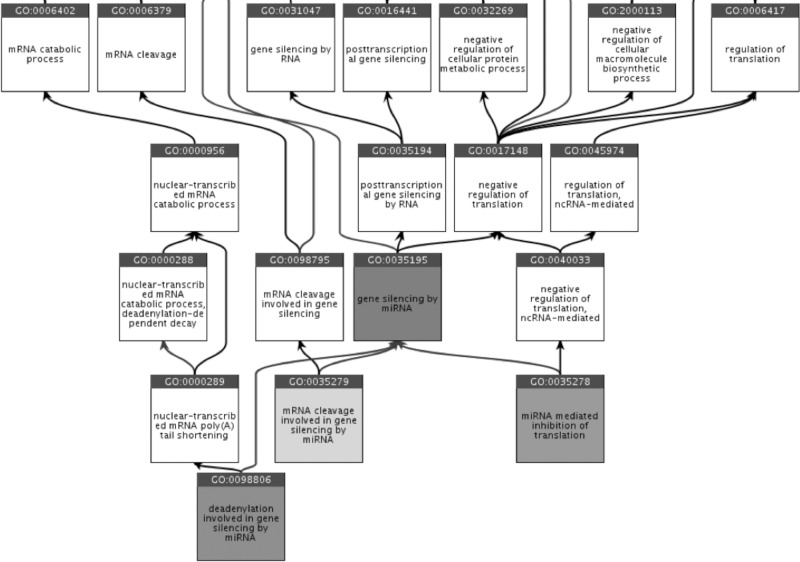
Ancestor chart from QuickGO showing the child terms of “gene silencing by miRNA” (GO:0035195, all highlighted with gray boxes), as well as some of the ancestor terms in this part of the ontology.

The GO and its associated annotations are continually evolving as biological knowledge increases and as curators focus on annotation and ontology development efforts in specific areas of biological interest (The Gene Ontology Consortium 2014). No dedicated effort had focused on the functional curation of miRNAs, or their biogenesis, and the ontology representing gene silencing by miRNA was consequently outdated and incomplete. For example, fundamental terms were absent from GO including a biological process (BP) term to describe one of the three common mechanisms of miRNA gene silencing—via the 3′ UTR by deadenylation. Figure 1 shows how these three common mechanisms are now represented in GO. In some cases, existing terms were defined based on outdated knowledge. For example, the BP term “gene silencing” (GO:0016458) was originally defined as “Any transcriptional or post-transcriptional process carried out at the cellular level that results in long-term gene inactivation”; referring here to inherited silencing of genes. However, miRNA-regulated gene silencing is not long-term inactivation, and after consultation the definition of this term was broadened to: “Any process carried out at the cellular level that results in either long-term transcriptional repression via action on chromatin structure or RNA mediated, post-transcriptional repression of gene expression.” The ontology was revised and updated in line with current knowledge prior to creation of the curation guidelines. So far, 11 new terms related to miRNA biogenesis and miRNA-dependent gene silencing have been created (Table 1) and a number of changes to ontology structure and term definitions have been made.

**TABLE 1.**
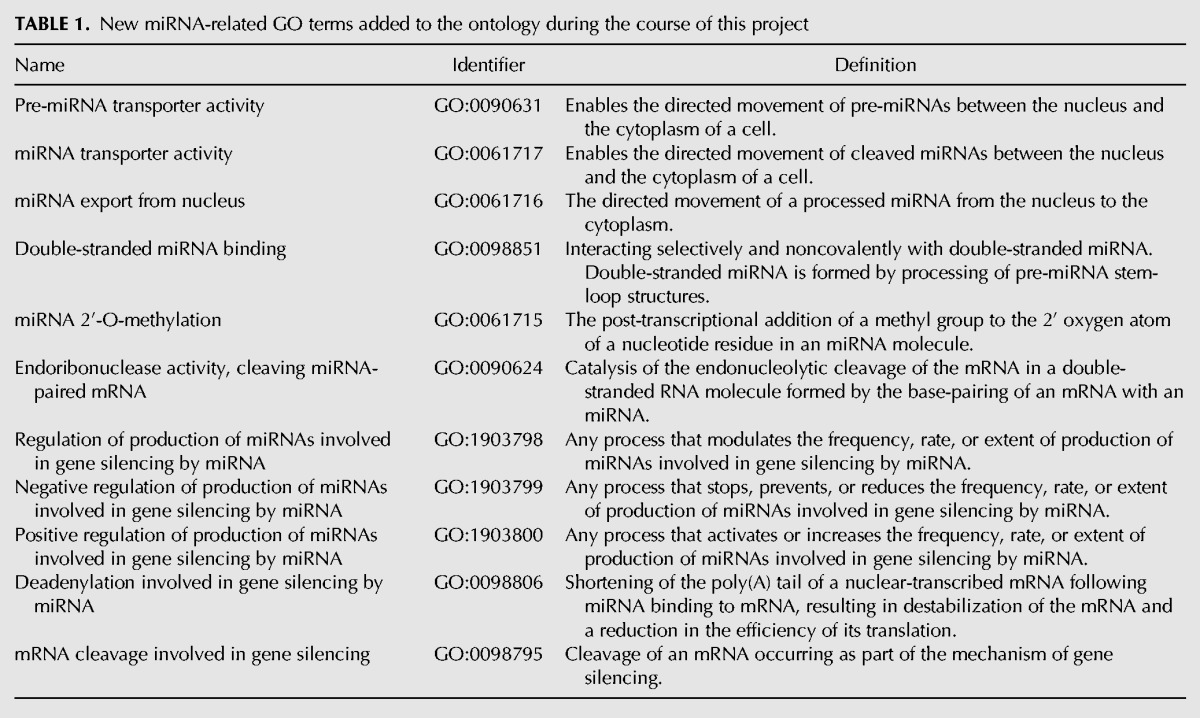
New miRNA-related GO terms added to the ontology during the course of this project

### Curation guidelines

#### Curating proteins involved in miRNA biogenesis

**FIGURE 2.**
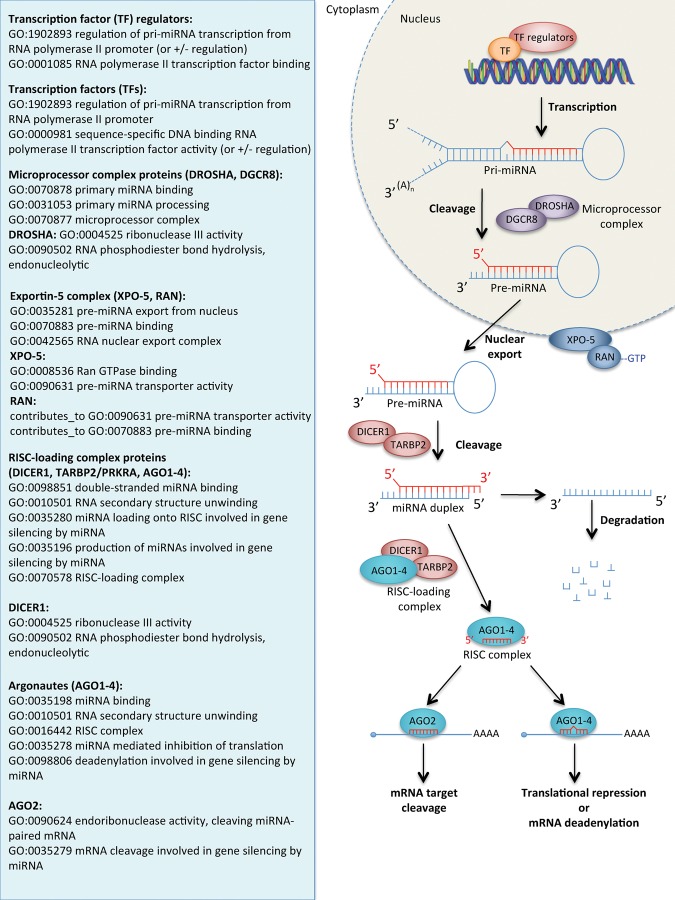
The canonical animal miRNA processing pathway (*right* panel) and the GO terms that are expected to be associated with the protein components of this pathway (*left* panel). Association of these GO terms will depend on the experimental evidence available; unrelated or more specific GO terms may be associated to these gene products if appropriate evidence is available. “contributes_to” is a qualifier used in GO annotation to indicate the entity annotated does not perform the molecular function in isolation, but as a member of a complex. Protein names: (DROSHA) Ribonuclease 3; (DGCR8) Microprocessor complex subunit DGCR8; (XPO-5) Exportin-5; (RAN-GTP) GTP-charged Ran GTPase; (DICER1) Endoribonuclease Dicer; (TARBP2) RISC-loading complex subunit TARBP2; (AGO) Argonaute.

**FIGURE 3.**
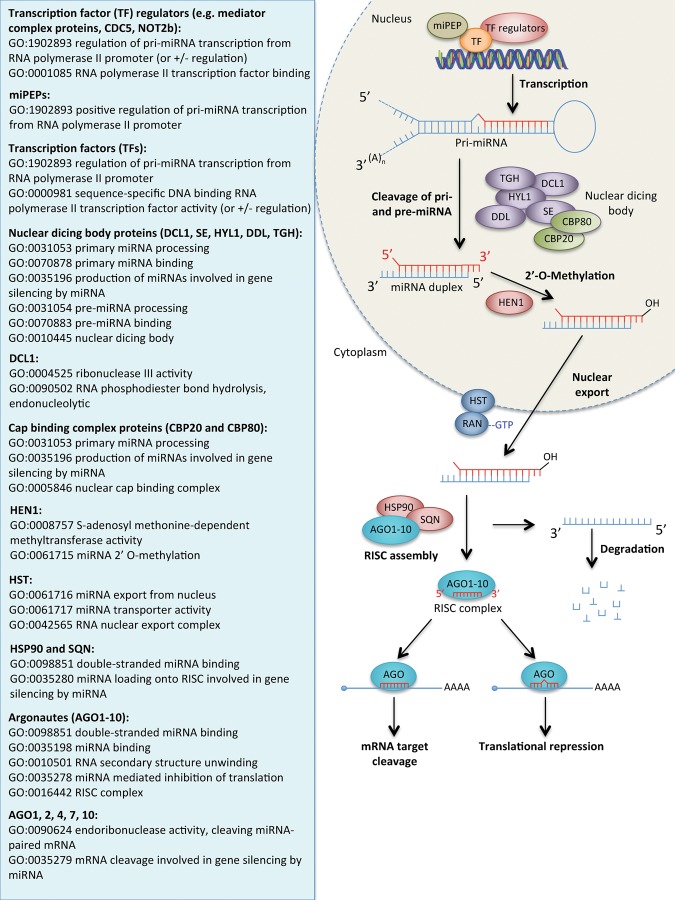
The canonical plant miRNA processing pathway (*right* panel) and the GO terms that are expected to be associated with the protein components of this pathway (*left* panel). Association of these GO terms will depend on the experimental evidence available; unrelated or more specific GO terms may be associated to these gene products if appropriate evidence is available. Protein names: NOT2B: NEGATIVE ON TATA-LESS 2B; CDC5: CELL DIVISION CYCLE 5; Mediator complex proteins (includes: MEDIATOR14, MEDIATOR20a, MEDIATOR20b, MEDIATOR20c, MEDIATOR21, MEDIATOR25); miPEP: miRNA encoded peptide; DCL1: DICER-LIKE 1; SE: SERRATE; HYL1: HYPONASTIC LEAVES 1; DDL: DAWDLE; TGH: TOUGH; CBP20: CAP BINDING COMPLEX PROTEIN 20; CBP80: CAP BINDING COMPLEX PROTEIN 80; HEN1: HUA ENHANCER 1; HST: HASTY; HSP90: HEAT SHOCK PROTEIN 90; SQN: SQUINT; AGO: ARGONAUTE.

The current knowledge representing miRNA biogenesis was reviewed and presented as a summary in the right-hand panels of Figure 2 (animal pathway) and Figure 3 (plant pathway). A description of the miRNA biogenesis pathway will not be covered here as it has been described extensively elsewhere, (e.g., Filipowicz et al. 2008; Axtell et al. 2011; Xie et al. 2015). Additionally, the pathway can be viewed interactively at Reactome (http://www.reactome.org/PathwayBrowser/#DIAGRAM=211000&ID=203927&PATH=74160) and the Rat Genome Database (http://rgd.mcw.edu/rgdweb/pathway/pathwayRecord.html?acc_id=PW:0000808). The GO terms applicable to each of the protein components or complexes to capture their role in miRNA biogenesis are presented in the left-hand panel of Figures 2 and 3. These terms do not provide comprehensive annotation of these proteins, many of which are also involved in other pathways.

#### Curating the role of miRNAs in gene silencing

**FIGURE 4.**
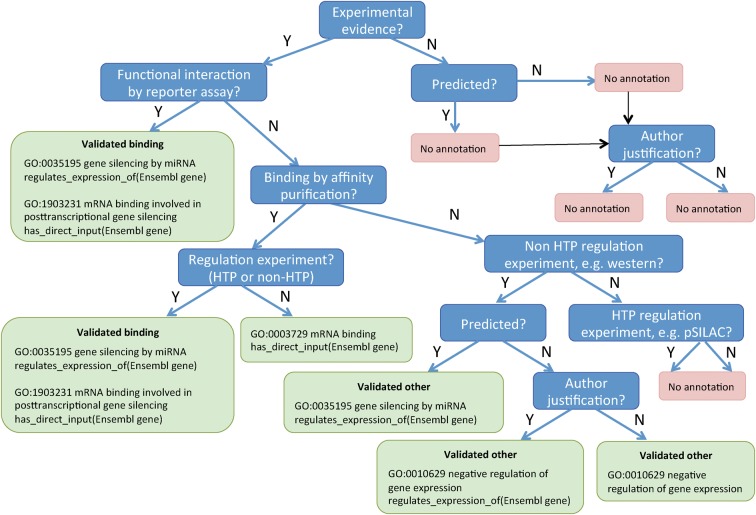
Decision tree for selecting the GO terms and annotation extensions used for capturing targets of miRNAs. The types of evidence in the blue boxes are described further in the online guidelines. A reporter assay, or an affinity purification together with an assay demonstrating an effect of the miRNA on mRNA levels, is sufficient to classify a target as “validated binding”; additional evidence that the target is predicted for the miRNA does not affect the annotation applied, therefore this option is not shown. Author justification means the author indicates why this mRNA is an expected target or shows an effect on an expected downstream process. (HTP) High-throughput method, (pSILAC) pulsed stable isotope labeling by amino acids in cell culture.

Depending on the evidence presented in a publication, there are several ways to represent the roles of an miRNA in gene silencing. To assist biocurators in selecting the correct GO terms for annotation, we have provided a decision tree for the different types of evidence presented by authors that can be used to support different gene silencing roles (Fig. 4). Briefly, if binding of the miRNA to the mRNA is demonstrated followed by a reduction in mRNA levels, for example, by showing that application of the miRNA to the target mRNA 3′ UTR fused to a luciferase reporter results in decreased reporter expression while application to a mutated form of the 3′ UTR has no effect (Clément et al. 2015), the miRNA should be annotated to the molecular function (MF) term “mRNA binding involved in post-transcriptional gene silencing by miRNA” (GO:1903231). If binding is not demonstrated but there is evidence of a reduction in the levels of an mRNA in response to an miRNA, for example, an experiment showing that application of a given miRNA results in decreased target mRNA levels as measured by qRT-PCR (e.g., see Maegdefessel et al. 2012), then either the BP term “negative regulation of gene expression” (GO:0010629) should be used (if the mRNA is not a predicted target) or “gene silencing by miRNA” (GO:0035195, if the mRNA is a predicted target). In each case, the target mRNA must be indicated within the annotation, as detailed in the annotation examples in the section “Curating the mRNA targets of miRNAs” and in Figure 4. In certain circumstances, when the authors have shown the exact mechanism of silencing, i.e., translational repression, deadenylation or mRNA cleavage, it is possible to use the child terms of the BP term “gene silencing by miRNA” as indicated in the following sections.

Translational repression. If there is sufficient evidence to show the miRNA is involved in translational repression of the mRNA target, then the GO BP term “miRNA-mediated inhibition of translation” (GO:0035278) should be used. For example, application of mouse miR-1 leads to a decrease in the protein level of histone deacetylase 4 (HDAC4), but has no effect on the level of mRNA encoding this protein (Chen et al. 2006).

mRNA deadenylation. The GO BP term “deadenylation involved in gene silencing by miRNA” (GO:0098806) should be used if there is sufficient evidence to show the miRNA is involved in deadenylation of the mRNA target. This GO term was associated with the *Drosophila* miRNA Let-7 based on experiments described by Wakiyama et al. (2007). In these experiments, the length of the poly(A) tail of a luciferase reporter mRNA containing six Let-7 target sequences was reduced to almost the same length as the nonadenylated control mRNA following treatment with Let-7, while a control containing mutated Let-7 sequences was unaltered.

mRNA cleavage. If there is sufficient evidence to show the miRNA is involved in cleavage of the mRNA target, the GO BP term “mRNA cleavage involved in gene silencing by miRNA” (GO: 0035279) should be used. For example, Wang and Guo (2015) detected a specific cleavage site within the mRNA of the *Arabidopsis* auxin responsive protein IAA28, by a 5′ RACE assay and subsequent sequencing, following application of *Arabidopsis* miRNA-847.

#### Curating the mRNA targets of miRNAs

One of the main criteria for an mRNA to be targeted by an miRNA is perfect or near-perfect complementarity to the 5′ end of the miRNA, the so-called “seed region” at positions 2 to 7, but this is not the only determinant (Betel et al. 2010). The other determinants used by computational algorithms to predict targeted mRNAs, with varying success, include sequence alignment and conservation, mRNA secondary structure analysis and calculation of hybridization energy (Grimson et al. 2007; Betel et al. 2008; Wang and El Naqa 2008). However, even if an mRNA is a predicted target of an miRNA, it is not certain that it will be a target in vivo. It is therefore important to distinguish between real targets and potential targets, which is only possible by experimental investigation. An increasing number of experimental methods are available for testing either the binding or the regulation aspects of miRNA:mRNA functional interaction, some of which are classified as high-throughput (HTP) (Thomson et al. 2011; Chou et al. 2015). To assist biocurators in deciding if the evidence for an miRNA:mRNA functional interaction is sufficient to create an annotation we have provided a list of commonly reported methodologies in the online miRNA curation guidelines. This includes a description of the method and whether it is sufficient on its own to demonstrate the binding and regulation aspects or whether additional experimental evidence is required. We recommend that in cases which combine two methods for demonstrating functional interaction, only one of these should be a HTP method as indicated on the list; as technologies improve this decision may be revisited. For example, a reporter assay alone is sufficient to demonstrate binding to and regulation of the mRNA, but a CLASH experiment (HTP) can only demonstrate binding of the miRNA:mRNA; therefore, additional non-HTP evidence demonstrating the regulation of the mRNA levels is also required, such as a qRT-PCR. For the purpose of determining how a target should be captured by GO annotation, we have defined three categories of mRNA target according to what evidence is available for the association as follows:

Predicted targets. A predicted target is an mRNA that has a 3′ UTR sequence predicted to contain the binding site(s) of the miRNA. Targets are predicted in silico using sophisticated algorithms (Ekimler and Sahin 2014; Afonso-Grunz and Müller 2015). Predicted targets are not captured as GO annotations but are used to inform the GO term that should be used for validated “other” targets (see below and Fig. 4).

Validated binding targets. A validated binding target is an mRNA that has undergone experimental investigation to determine that the miRNA both binds to and regulates the expression of the mRNA. The most applicable evidence is a reporter assay, where the miRNA is combined with a reporter-fused 3′ UTR of the mRNA and altered levels of reporter expression are observed (Clément et al. 2015). Also acceptable is an assay demonstrating physical interaction between the miRNA:mRNA, e.g., affinity purification, together with an assay demonstrating that the miRNA alters the levels of either the mRNA (e.g., qRT-PCR) or protein (e.g., Western blot) (Dangwal et al. 2012). It must be noted, however, that these methods only provide an indication that the miRNA is able to bind the mRNA not that it will bind in a physiological context, where there may not be coincident expression of the miRNA and mRNA and also where the concentrations and stoichiometry of the miRNA and mRNA may be different.

Validated “other” targets. A validated “other” target is an mRNA that has undergone experimental investigation to demonstrate miRNA regulation of the target, but has not conclusively been shown to bind to the miRNA. Assays that demonstrate this include western blot, qRT-PCR, or pSILAC (Dangwal et al. 2012).

This classification enables biocurators to decide which GO terms and associated information can be applied to the miRNA:mRNA pairing. To assist biocurators further, the decision tree (Fig. 4) uses experimental assays as the deciding factors for building an accurate annotation. To maintain a high-quality, reliable resource for miRNA target data, we only curate experimentally validated targets from peer-reviewed publications.

Annotation extensions provide a mechanism to associate additional information with a GO term (Huntley et al. 2014), and are used to capture miRNA target genes (illustrated in Fig. 4). Depending on the available experimental data, with GO annotations we can distinguish between targets that are bound by the miRNA and those where there is insufficient evidence to demonstrate direct binding. An mRNA target that is experimentally demonstrated to bind the miRNA, leading to its reduced expression (*validated binding*) can be annotated as follows:^16^

**Annotation 1**
Object: human miR-21 (RNACentral:URS000039ED8D_9606)
GO term: gene silencing by miRNA (GO:0035195)
Annotation Extension: *regulates_expression_of* human *SPRY2* gene (Ensembl:ENSG00000136158)
Reference: PubMed:23239100

**Annotation 2**
Object: human miR-21 (RNACentral:URS000039ED8D_9606)
GO term: mRNA binding involved in post-transcriptional gene silencing (GO:1903231)
Annotation Extension: *has_direct_input* human *SPRY2* gene (Ensembl:ENSG00000136158)
Reference: PubMed:23239100

Combined, these annotations mean that human miR-21 can bind the *SPRY2* mRNA causing silencing of the gene's expression.

An mRNA target that is experimentally demonstrated to be a target of the miRNA, but for which there is insufficient evidence to show that it binds the miRNA (*validated other*), can be annotated as follows:

Object: human miR-200b (RNACentral:URS000014D9C1_9606)
GO term: gene silencing by miRNA (GO:0035195)
Annotation Extension: *regulates_expression_of* human *PTPN12* gene (Ensembl:ENSG00000127947)
Reference: PubMed:16762633

This annotation means that human miR-200b silences expression of *PTPN12* but the molecular mechanism of silencing has not been established. As noted earlier, if the exact mechanism of silencing is shown experimentally the child terms of “gene silencing by miRNA,” e.g., “deadenylation involved in gene silencing by miRNA,” should instead be used (Fig. 1).

#### Capturing the context of gene silencing

Each miRNA can have hundreds of predicted targets depending on the cell or tissue type, or developmental stage of the organism. Therefore, the miRNA may have different available targets and different biological roles depending on the context of its expression. We can capture this contextual information in the GO annotations using annotation extensions. Mouse miR-29b, for example, was shown to reduce the mRNA levels of elastin through use of an miRNA mimic that increased levels of miR-29b in smooth muscle cells, a cell type known to express miR-29b (Zampetaki et al. 2014). The GO annotation created is:

Object: mouse miR-29b (RNACentral:URS000024463E_10090)
GO term: gene silencing by miRNA (GO:0035195)
Annotation Extension: *regulates_expression_of* mouse *ELN* gene (Ensembl:ENSMUSG00000029675), *occurs_in* smooth muscle cell (CL:0000192)
Reference: PubMed:25201911

This annotation is interpreted as: mouse miR-29b is involved in gene silencing of mouse *ELN* in smooth muscle cells. Providing these contextual details within the GO annotation will assist researchers wishing to perform cell- or tissue-specific network analyses.

#### Capturing the downstream effects of gene silencing

A major use of GO is large-scale analysis of gene function, for example GO term enrichment. Analysis of miRNA data sets will be improved, therefore, by curating the consequential effects of specific miRNAs on the cell or organism, i.e., the physiological processes regulated by the silencing event. By way of example, Castaldi et al. (2014) demonstrated that mouse miR-133a could silence expression of the adenylate cyclase *ADCY6* gene. The authors went on to show the effect that this silencing had on cardiac muscle cells: the enzyme activity of the adenylate cyclase and rate of cAMP accumulation were reduced. This observation is captured in the following annotation:

Object: mouse miR-133a (RNACentral:URS00004C9052_ 10090)
GO term: negative regulation of adenylate cyclase activity (GO:0007194)
Annotation Extension: *occurs_in* cardiac muscle cell (CL: 0000746)
Reference: PubMed:24807785

This annotation is interpreted as: mouse miR-133a is involved in the attenuation of adenylate cyclase activity in cardiac muscle cells. Note: Because the authors measured total adenylate cyclase activity, not specifically ADCY6, it is not possible to add ADCY6 as the target of the regulation.

It is important to note that if the authors state that the miRNA is being expressed in cells/tissues where it would not normally be expressed, the experiment is not physiologically relevant information—miRNAs are known to have different targets in different cell and tissue types (Zhu et al. 2011)—so it would not be appropriate to create an annotation.

The inclusion of such biologically relevant effects of miRNA gene silencing in the GO annotation data set will enable researchers to perform functional enrichment on the annotations that are associated directly with miRNAs, allowing more reliable and significant interpretations of large-scale data.

### Evidence codes

Evidence codes are used in GO annotation to indicate the type of evidence that is available in a paper to support the association of a GO term with a gene product (Balakrishnan et al. 2013). The use of evidence codes for curation of miRNAs follows the GOC guidelines (http://geneontology.org/page/guide-go-evidence-codes). Generally, there are two types of experiments used when investigating the role of an miRNA: (i) increasing the amount of miRNA, or (ii) decreasing the amount or effectiveness of an miRNA. The evidence code used will therefore be based on which type of modulation is applied (see van Rooij et al. 2008 for a review of miRNA modulations): (i) When increasing the amount of miRNA, e.g., by using a pre-miRNA, the miRNA should be annotated using “inferred from direct assay” (IDA); this is because the sequence is unchanged and these experiments can provide valuable information about the normal, in vivo function of the miRNA. If an over-expression of an miRNA clearly results in a gain-of-function that is not physiologically relevant, this is not curated. (ii) When decreasing the amount of miRNA or inhibiting its activity, e.g., by using an antagomir or mutating the sequence, the miRNA should be annotated using the “inferred from mutant phenotype” (IMP) evidence code; this is because the cellular effect of “disturbing” the normal functioning of the miRNA is used to make an inference about the normal, in vivo function of the miRNA.

### Inferring knowledge from other species

In the absence of experimental data for a particular species, a common practice in GO is to make an annotation based on sequence similarity or orthology using the evidence codes inferred from structural or sequence similarity (ISS) or inferred from sequence orthology (ISO) (Balakrishnan et al. 2013). Caution must be used by any curator wishing to infer knowledge about the function of an miRNA in one species from experimental data for an miRNA from another species. This is because even slight variations in the sequences of the mRNA or miRNA can cause loss of complementarity and disruption of the interaction, as demonstrated when using a mutated form of either the miRNA or mRNA in a standard reporter assay used to validate targets of miRNAs (Clément et al. 2015). Individual annotation groups will have different policies with respect to whether or not annotations are transferred to miRNAs from other species.

### Summary

MiRNA annotations should minimally aim to capture (i) the miRNA's main role in gene silencing and its target(s), e.g., “gene silencing by miRNA” with the target gene in the annotation extension field, and (ii) the effect of silencing the target mRNA, e.g., “negative regulation of adenylate cyclase activity.”

## DISCUSSION

Resources for analyzing miRNA functional information are currently restrictive for downstream analysis. Here we describe the curation of functional data to support miRNA research, providing scientists access to high-quality, reliable data with which to inform their hypotheses and plan future experiments. Over time, these guidelines applied to miRNA annotation will provide miRNA target information that is easy to navigate to identify the experimentally validated targets of a particular miRNA. Contextual information provides experimentally verified links between miRNAs and other physiological information that is crucial for accurate analysis of pathway and network data (Khatri et al. 2012). Annotation of regulated processes directly to the miRNA will enable relevant functional analyses, such as GO term enrichment. Just as GO protein annotation strategies have been applied to link pathway databases such as Reactome to the GO framework, the miRNA annotation strategy outlined here will enable miRNA annotation to be similarly utilized, e.g., for the Reactome Pre-NOTCH transcription and translation pathway http://www.reactome.org/PathwayBrowser/#/R-HSA-1912422&SEL=1912408&PATH=R-HSA-162582,R-HSA-157118. These guidelines are publicly available and for use by any scientist wishing to describe the roles of miRNAs and submit annotations to the GO Consortium. Adoption of these guidelines will allow biocurators to build valuable resources useful to the wider scientific community. We will continue refining these guidelines accordingly as new knowledge is obtained.

We encourage contributions of miRNA GO annotations for inclusion into the GO Consortium database; contact the GO helpdesk (http://geneontology.org/form/contact-go) for more information about the tools available to support miRNA annotation and the required format of submitted annotations.

## MATERIALS AND METHODS

### Functional annotation

We describe the functional annotation of miRNAs and proteins using the GO vocabulary. The workflow for the general functional curation of gene products is published elsewhere and provides a useful guide for basic GO annotation techniques (Balakrishnan et al. 2013). The current article describes only the key aspects of curating miRNAs, the full miRNA curation guidelines are available on the GO Consortium wiki (http://wiki.geneontology.org/index.php/MicroRNA_GO_annotation_manual).

### Ensuring correct representation of current knowledge by expert biocuration

To ensure that a reliable source of functional data is maintained for miRNA research, we recommend that curation of miRNAs is based on experimental data from peer-reviewed journals. Even when using such high-quality experimental data, the translation of biological knowledge into GO annotation can be challenging, especially in such a new and rapidly evolving field of research. During this project, we have updated the ontology with current knowledge—where necessary working with experts in the field to establish correct definitions and placement of terms within the ontology. Using the improved ontology we have begun to fill in gaps in annotations and to correct any errors that may exist.

### Defining the scope of the guidelines

To give a full picture of regulation of gene expression by miRNAs, from their initial transcription to their roles in gene silencing, we first make recommendations for the curation of the protein components of the canonical miRNA biogenesis pathways in animals and plants. We then proceed to provide recommendations for curation of the miRNAs’ roles in gene regulation. As research progresses, alternative mechanisms of miRNA regulation are being discovered (Filipowicz et al. 2008; Hausser et al. 2013; Wilczynska and Bushell 2014; Zhang et al. 2014). Here we focus on the best-studied mechanism: the effect of miRNAs on gene silencing via the 3′ UTR of mRNAs. We curate only miRNAs that are experimentally verified as involved in gene silencing and include their validated gene targets, ensuring we maintain a high-quality and reliable resource. We capture the experimentally demonstrated effect of the specific genes’ silencing on the cell or organism by annotating the miRNA directly with the relevant GO BP terms, something that has not been provided in other resources to date but that is critical information for unbiased functional analyses. We describe how contextual data for the roles of miRNAs are captured, including cell or tissue types. Further details on using annotation extensions to capture contextual data are available in Huntley et al. (2014).

### Database identifiers

The recommended database identifier for miRNAs in GO annotation are those provided by RNAcentral (The RNAcentral Consortium 2015), e.g., URS000039ED8D_9606 for human miR-21. Any stable identifier, e.g., Model Organism Database or Ensembl, may be used for the mRNA target gene. Here we have used Ensembl gene identifiers for the regulated gene, e.g., Ensembl:ENSG00000136158 for human *SPRY2* gene.

## Footnotes

16 Note that in actual GO annotations, only the database identifiers are used for curated entities and GO terms, not textual names included here for illustration purposes (see Materials and Methods for more details).
